# Cardiac-enriched BAF chromatin-remodeling complex subunit Baf60c regulates gene expression programs essential for heart development and function

**DOI:** 10.1242/bio.029512

**Published:** 2017-11-28

**Authors:** Xin Sun, Swetansu K. Hota, Yu-Qing Zhou, Stefanie Novak, Dario Miguel-Perez, Danos Christodoulou, Christine E. Seidman, J. G. Seidman, Carol C. Gregorio, R. Mark Henkelman, Janet Rossant, Benoit G. Bruneau

**Affiliations:** 1 Program in Developmental and Stem Cell Biology, The Hospital for Sick Children, Toronto, ON, M5G 1X8 Canada; 2 Department of Molecular Genetics, University of Toronto, Toronto, ON M5S 1A8 Canada; 3 Gladstone Institutes, San Francisco, CA, 94158 USA; 4 Roddenberry Center for Stem Cell Biology and Medicine at Gladstone, Gladstone Institutes, San Francisco, CA 94158, USA; 5 The Mouse Imaging Centre, The Hospital for Sick Children, Toronto, ON, M5G 1X8 Canada; 6 Department of Cellular and Molecular Medicine, University of Arizona, Tucson, AZ 85724, USA; 7 Department of Genetics, Harvard Medical School, Boston, MA 02115, USA; 8 Howard Hughes Medical Institute, Brigham and Women's Hospital, Boston, MA 02115, USA; 9 Department of Medical Biophysics, University of Toronto, Toronto, ON M5S 1A8 Canada; 10 Department of Pediatrics, University of California, San Francisco, CA 94143, USA; 11 Cardiovascular Research Institute, University of California, San Francisco, CA 94158, USA

**Keywords:** Chromatin remodeling, Embryo, Gene regulation, Heart

## Abstract

How chromatin-remodeling complexes modulate gene networks to control organ-specific properties is not well understood. For example, *Baf60c* (*Smarcd3*) encodes a cardiac-enriched subunit of the SWI/SNF-like BAF chromatin complex, but its role in heart development is not fully understood. We found that constitutive loss of *Baf60c* leads to embryonic cardiac hypoplasia and pronounced cardiac dysfunction. Conditional deletion of *Baf60c* in cardiomyocytes resulted in postnatal dilated cardiomyopathy with impaired contractile function. *Baf60c* regulates a gene expression program that includes genes encoding contractile proteins, modulators of sarcomere function, and cardiac metabolic genes. Many of the genes deregulated in *Baf60c* null embryos are targets of the MEF2/SRF co-factor Myocardin (MYOCD). In a yeast two-hybrid screen, we identified MYOCD as a BAF60c interacting factor; we showed that BAF60c and MYOCD directly and functionally interact. We conclude that Baf60c is essential for coordinating a program of gene expression that regulates the fundamental functional properties of cardiomyocytes.

## Abstract (precis)

**Summary:** Genetic deletion of *Baf60c* leads to embryonic cardiac hypoplasia and dysfunction. Baf60c coordinates a program of gene expression that regulates the fundamental functional properties of cardiomyocytes.

## INTRODUCTION

Transcription factor networks control cardiac morphogenesis and cell specification (Bruneau, 2013; Evans et al., 2010), including the coordinated regulation of genes encoding the proteins involved in sarcomere function (Creemers et al., 2006; Niu et al., 2008). While undergoing complex morphogenetic changes, the developing heart supports embryonic circulation. The contractile function of the heart adapts quickly to the dramatic changes in circulation that occur after birth and, subsequently, must adapt to fluctuating physiology and stress. The transcriptional regulation of cardiac gene expression continues during postnatal heart growth and cardiomyocyte maintenance (Huang et al., 2009; Oka et al., 2006).

Chromatin-remodeling complexes are critical regulators of cardiac gene expression, in many cases modulating the activity of DNA-binding transcription factors (Chang and Bruneau, 2012). For example, histone deacetylases (HDACs) and bromodomain-containing factors have important roles in cardiac gene regulation and remodeling, and have been proposed as potential drug targets (Anand et al., 2013; McKinsey, 2012). BRG1/BRM-associated factor (BAF) complexes are ATP-dependent chromatin remodeling complexes related to the yeast SWI/SNF complex, and are indispensable for mammalian development (Hota and Bruneau, 2016). BAF complexes orchestrate many aspects of heart development, and genetically interact with cardiac transcription factors to finely modulate cardiac gene expression (Hang et al., 2010; Takeuchi et al., 2011). Combinatorial assembly of different polymorphic subunits can generate hundreds of potential BAF complexes, and offer precise control of developmental processes (Chang and Bruneau, 2012; Ho and Crabtree, 2010). BAF60c (also known as SMARCD3) is a polymorphic subunit of the BAF complex, which is expressed preferentially in the developing heart (Lickert et al., 2004). *In vivo* RNAi knockdown in mouse embryos suggested that *Baf60c* is essential for embryonic heart development (Lickert et al., 2004), and together with the cardiac transcription factors TBX5, NKX2-5 and GATA4, BAF60c can induce non-cardiac mesoderm to differentiate into cardiomyocytes (Lou et al., 2011; Takeuchi and Bruneau, 2009).

In this study, we examined the role of *Baf60c* in embryonic and postnatal heart development in a *Baf60c* conditional knockout mouse line. We showed that *Baf60c* is essential for cardiac growth and cardiomyocyte function at several stages of embryonic development, by regulating broad networks of genes encoding proteins essential for function of the contractile apparatus. Many of the dysregulated genes are targets of the MEF2 co-factor MYOCD, and we identified MYOCD as a BAF60c-interacting protein. Our work shows that *Baf60c* is an important modulator of the fundamental program of gene expression essential for cardiac structure and function.

## RESULTS

### Construction of *Baf60c* conditional knockout mouse line

**Fig. 1.**
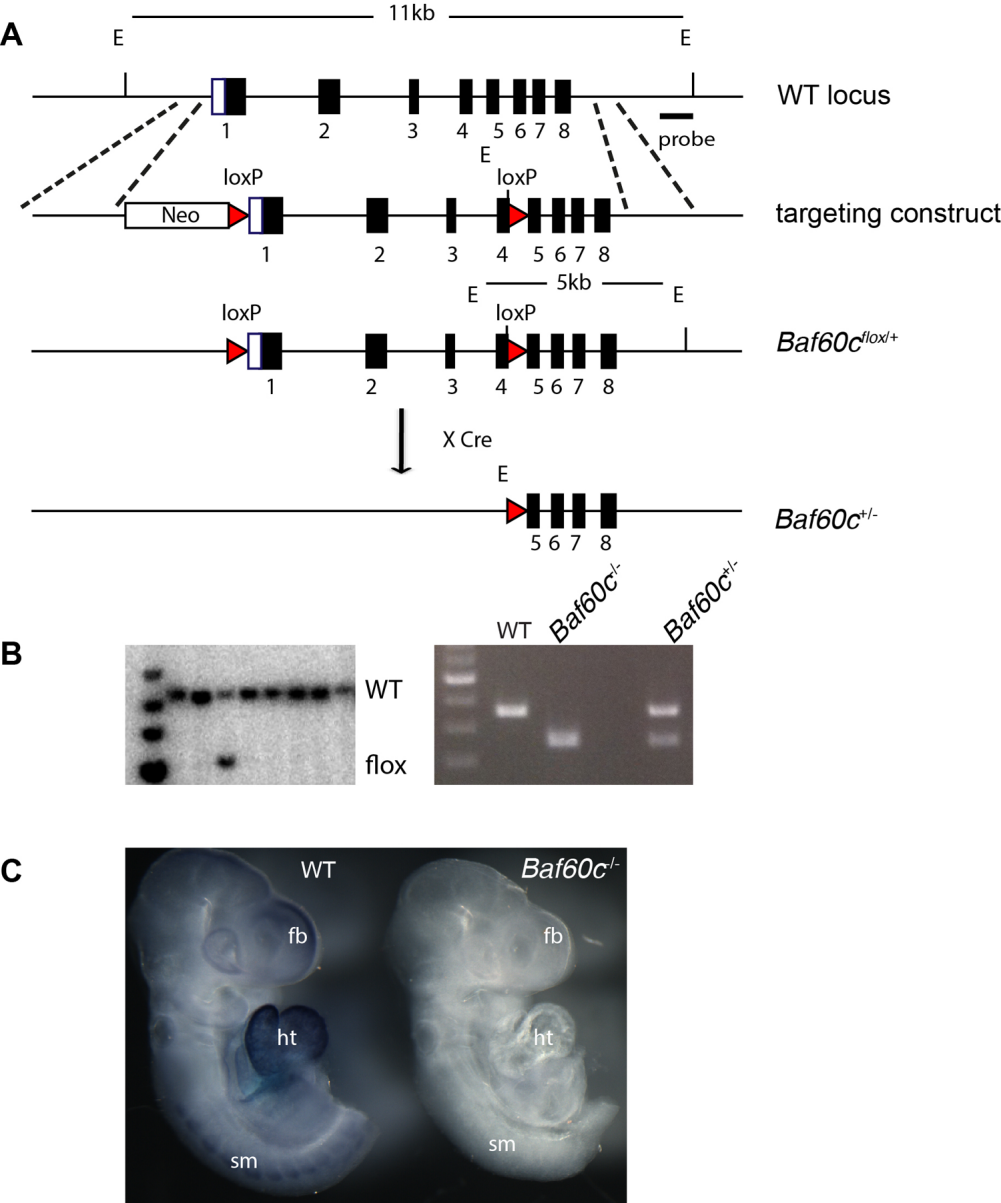
**Construction of *Baf60c* knockout mouse line.** (A) Schematic representation of targeting DNA introduced into wild-type (WT) *Baf60c* locus. Correctly targeted ES cells were identified with probe located outside of the homologous arm. Cre-mediated excision removed exons 1–4 and resulted in *Baf60c^+/−^.* (B) Left: Southern blot of digested ES cell DNA with an external probe outside of the targeting DNA. WT and targeted band size are as described in A. Right: genotype PCR showing the band size differences of WT, *Baf60c^−/−^* (KO) and heterozygous *Baf60c^+/−^*. (C) Whole-mount *in situ* hybridization using full-length Baf60c probe detected no signals in genotyped homozygous Baf60c^−/−^ embryos (*n*>3), indicating complete deletion. E, EcoRI; fb, forebrain; ht, heart; sm, somites.

*Baf60c* is expressed at embryonic day (E)7.5 in the early cardiac precursors of the cardiac crescent, and its expression is maintained throughout development in the myocardium (Lickert et al., 2004). To understand the function of *Baf60c* at different developmental stages, we developed a conditional allele of *Baf60c* in the mouse. A targeting construct with a pair of loxP sites flanking exon 1–4 was introduced into embryonic stem (ES) cells (Fig. 1A). Transgenic mice generated from the targeted ES cells (*Baf60c^flox^*^/+^) had normal phenotypes and lifespans and, thus, were treated as wild type. By crossing them with pCAGGS-Cre mice, which constitutively express Cre recombinase, exons 1–4 of *Baf60c* were deleted to generate *Baf60c*^+/−^ mice (Fig. 1A). No obvious defects were observed in *Baf60c*^+/−^ mice. Homozygous null *Baf60c*^−/−^ embryos were recovered at E9.5 (Fig. 1B), and by whole mount *in situ* hybridization, no *Baf60c* mRNA was detectable in *Baf60c^−^*^/*−*^ embryos (Fig. 1C).

### *Baf60c* deletion results in a hypoplastic heart and embryonic demise

**Fig. 2.**
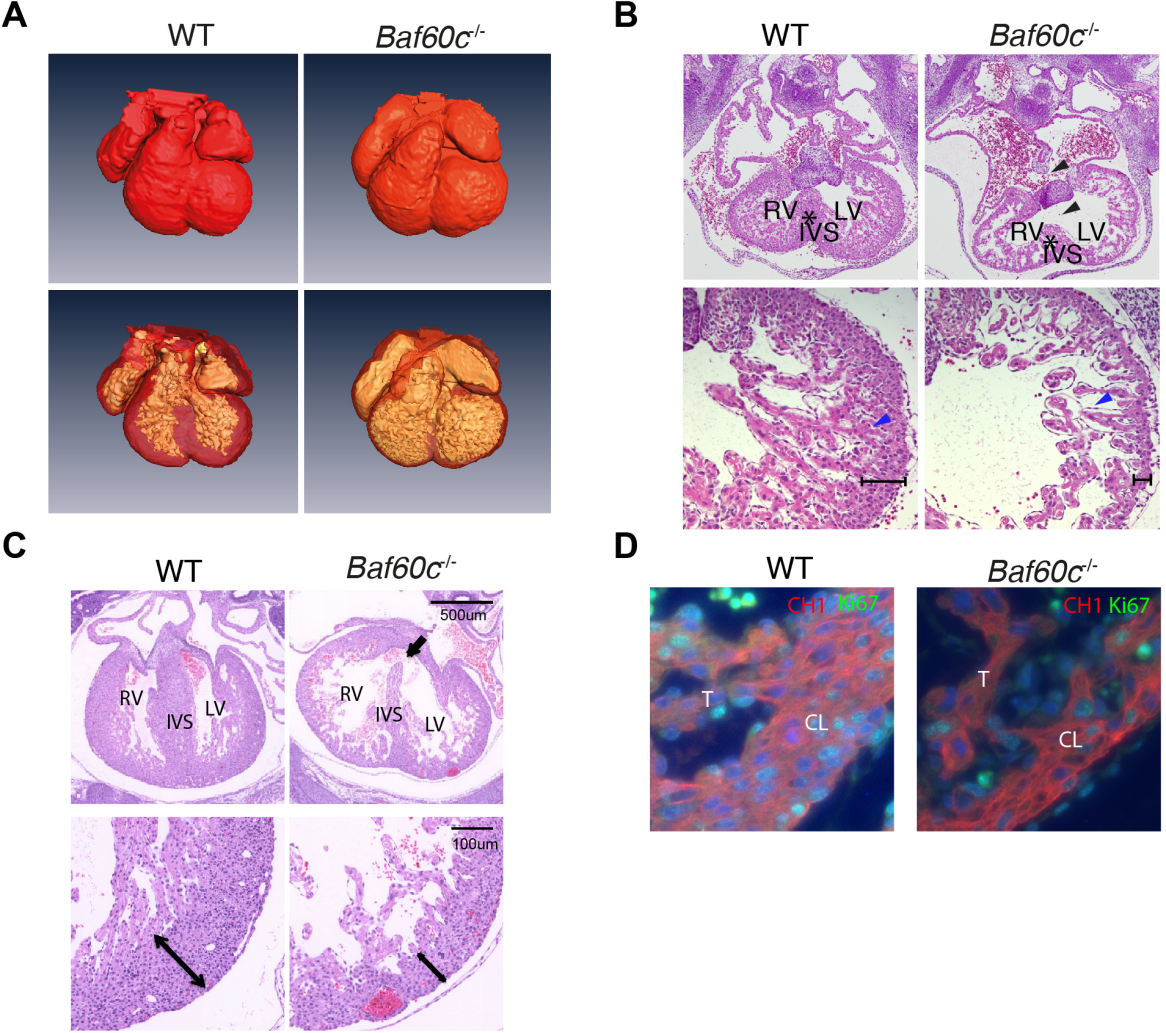
***Baf60c* deletion results in a hypoplastic embryonic heart.** (A) At E12.5, *Baf60c*^−/−^ embryonic hearts have similar outer dimensions as the wild type (WT), but the ventricle chambers are expanded and ventricle walls are thinner as observed by rendered OPT images. (B,C) Transverse sections and HE staining of E11.5 (B) and few surviving mixed background E14.5 (C) embryonic hearts. The *Baf60c^−^*^/*−*^ hearts show incomplete inter-ventricular septum formation (star) and have VSDs (black arrows or arrowheads), thinner ventricle walls (brackets) and disorganized and reduced trabeculation (blue arrowheads), compared to WT strains. (D) Ki67 staining detects fewer proliferating cardiomyocytes in E12.5 *Baf60c^−^*^/*−*^ heart than in WT. Red, CH1 anti tropomyosin; green, Ki67. CL, compact layer; T, trabeculae; RV, right ventricle; IVS, interventricular septum; LV, left ventricle.

*Baf60c^−^*^/*−*^ embryos were recovered alive and with roughly normal morphology at different stages of timed pregnancies until E12.5–E14.5. At E14.5, most *Baf60c^−^*^/*−*^ embryos were dead, with broad regions of hemorrhage. Backcrossing into C57Bl/6 for 10 generations led to a more consistent phenotype, with survival only until E12.5–13.5. To determine the cause of embryonic death and to identify potential cardiac phenotypes, *Baf60c^−^*^/*−*^ embryos were harvested for histological analysis. Optical projection tomography showed that mixed background E12.5 *Baf60c^−^*^/*−*^ embryonic hearts had dilated inner chambers and underdeveloped interventricular septa (Fig. 2A). At E11.5, *Baf60c^−^*^/*−*^ C57Bl/6 embryonic hearts had a more severe and penetrant phenotype, with a thin compact layer and fewer or less well-developed trabeculae (Fig. 2B), impaired atrioventricular cushion formation, and reduced atrial septum growth. In the few surviving E14.5 *Baf60c^−^*^/*−*^ mixed background embryos, ventricular free walls were much thinner than wild type (Fig. 2C), and the interventricular septum was disorganized, leading to ventricular septal defects. Based on the intrinsic cardiac phenotypes, we conjectured that circulatory failure and hemorrhage were the result of impaired cardiac function of *Baf60c^−^*^/*−*^ embryos.

To identify the possible cause of cardiac hypoplasia in *Baf60c* knockouts, proliferation of cardiomyocytes was assessed by staining with Ki67 antibody. Immunostaining detected fewer Ki67+ cardiomyocytes in E12.5 *Baf60c^−^*^/*−*^ ventricles than in wild type (Fig. 2D). Quantitation confirmed that, in wild-type (WT) hearts, there were 32±9% Ki67+ ventricular cardiomyocytes, and in *Baf60c^−^*^/*−*^ hearts, 25±5% were positive (*n*=4; *P*<0.05 by unpaired two-tailed *t*-test). There was no evidence of increased apoptosis.

The embryonic heart begins to pump blood from the linear heart tube stage onwards, and its contractile function is essential for fetal life. To determine if cardiac function was affected by *Baf60c* deletion, we used high frequency ultrasound echocardiography (Zhou et al., 2002) to evaluate contractile parameters of E13.5 mixed background embryos *in utero* (Table 1). No regurgitation between atria and ventricles was observed in *Baf60c^−^*^/*−*^ embryos, which indicates that cardiac valves had formed and were fully functional. However, the left ventricle fraction shortening (LVFS) of *Baf60c^−^*^/*−*^ hearts was reduced, suggesting impaired systolic function. The inter-ventricular septal fractional thickening (IVSFT) was lower than in the hearts of WT and *Baf60c^+^*^/*−*^ embryos, indicating reduced myocardial contraction. The E:A ratios of *Baf60c^−^*^/*−*^ hearts for both the left and right ventricles were also significantly increased. This may indicate impaired cardiac relaxation (Zhou et al., 2003). Overall, echocardiography showed that loss of *Baf60c* affected the morphology and dimensions of the heart and, concomitantly, its contractile function.

**Table 1.**
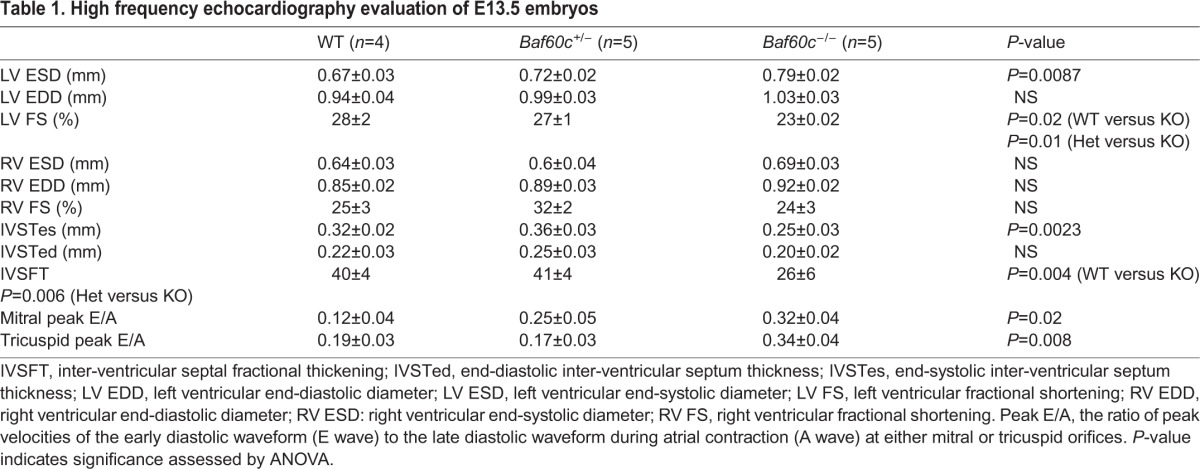
**High frequency echocardiography evaluation of E13.5 embryos**

**Fig. 3.**
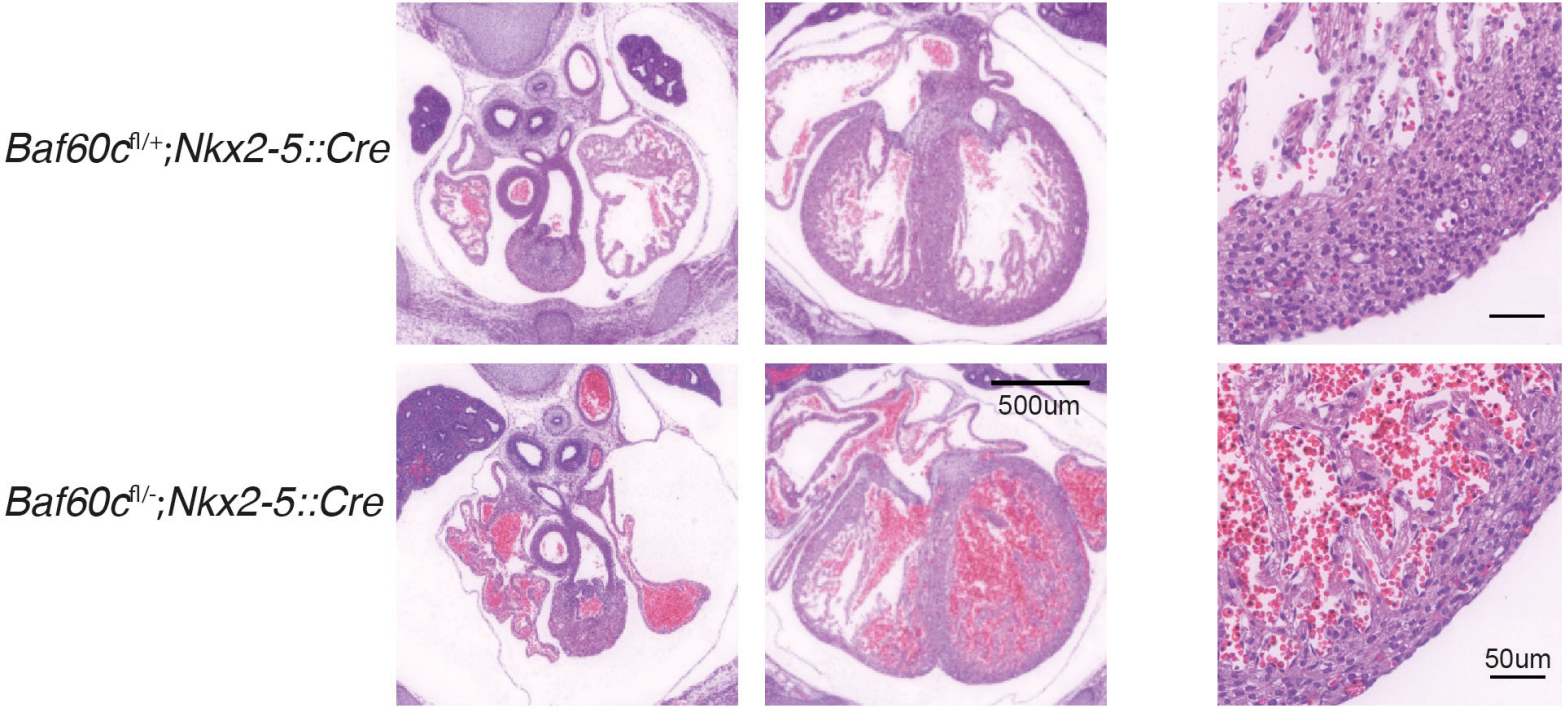
**Deletion of Baf60c with *Nkx2-5^Cre^*.** HE staining of transverse sections of E14.5 mouse heart shows thinner myocardium, reduced trabeculation, and ventricular septation defects.

Since *Baf60c* is expressed at sites outside the developing heart, such as extraembryonic tissues and neural tube, we assessed the tissue specificity of the *Baf60c^−^*^/*−*^ phenotype by crossing *Baf60c^flox^*^/*flox*^ mice with *Nkx2-5::Cre* mice. *Nkx2-5::Cre* deletes loxP-flanked DNA from E8.0 in all cardiac precursors (Moses et al., 2001). No live *Baf60c^flox^*^/−^;*Nkx2-5::Cre* embryos were recovered after E14.5. By E14.5 *Baf60c^flox^*^/−^;*Nkx2-5::Cre* embryo ventricle free walls and ventricular septum were much thinner than in littermate controls (Fig. 3). The ventricular septum was also poorly organized. This hypoplastic cardiac phenotype of *Baf60c^flox^*^/−^;*Nkx2-5::Cre* embryos phenocopies the least affected E14.5 *Baf60c*^−/−^ embryos, and suggests that the cardiac defects of the *Baf60c* knockout is a direct effect of Baf60c absence in the heart, instead of an indirect effect from *Baf60c* loss in other tissues like neural tube and somites. Indeed, no other embryonic defects were noted in *Baf60c^flox^*^/−^;*Nkx2-5::Cre* embryos. The constitutive null phenotype thus likely reflects primary loss of *Baf60c* in the developing heart, but as it is comparatively more severe might also reflect an earlier function in precursors that do not yet express *Nkx2-5* (Devine et al., 2014).

### Loss of Baf60c in cardiomyocytes results in postnatal cardiomyopathy

**Fig. 4.**
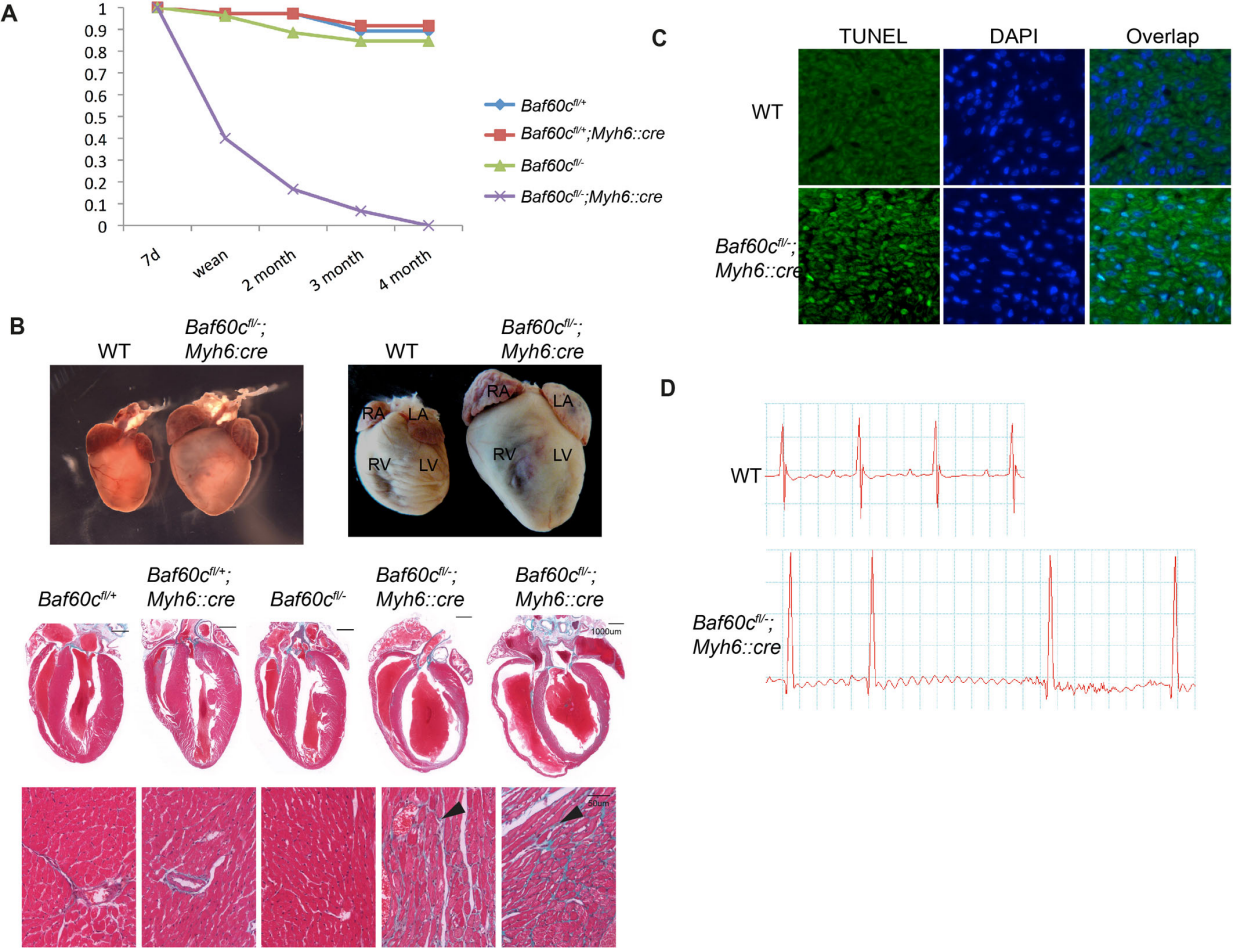
**Deletion of *Baf60c* in myocardium results in dilated chambers and impaired cardiac function.** (A) *Baf60c^fl/−^;Myh6::Cre* mice all die before 4 months of age. Fraction of remaining mice of each genotype is graphed. Initial number was 129 (37 flox/+, 36 flox/+;Cre, 26 flox/-, 30 flox/-;Cre). (B) *Baf60c^fl/−^;Myh6::Cre* mice have enlarged hearts and dilated chambers, as shown with whole-mount (top panel) and four-chamber view sections (middle panel). Left panel: P10. Right panel: 8-week hearts. Masson trichrome staining detects fibrosis in ventricle myocardium (bottom panels, arrowheads). (C) *Baf60c^Myh6KO^* myocardium have high levels of apoptosis. Green, TUNEL; blue, DAPI. (D) Representative electrocardiogram of adult WT and *Baf60c^fl/−^;Myh6::Cre* mice.

After birth, heart development switches from cell proliferation to hypertrophic growth. The structure and physiological function of the myocardium undergo a series of changes to adapt to a new hemodynamic environment. We deleted *Baf60c* in the myocardium at later developmental stages by crossing the *Baf60c^flox^*^/*flox*^ allele with *Myh6::Cre* (Agah et al., 1997). This manipulation bypassed the embryonic lethality of the constitutive deletion, as *Baf60c^flox^*^/−^;*Myh6::Cre* (*Baf60c^Myh6KO^*) mice were born alive and showed no obvious morphological changes before postnatal day (P)7. After P7, some of the *Baf60c^Myh6KO^* pups had delayed growth, compared with their littermates, and died before weaning. Other *Baf60c^Myh6KO^* mice survived after weaning without obvious morphological defects, but, at 4–6 weeks, exhibited symptoms of heart failure, including weight loss, reduced activity level, hunched back and labored breath. The remaining *Baf60c^Myh6KO^* mice appeared normal, but died suddenly. All *Baf60c^Myh6KO^* mice died before 4 months of age (Fig. 4A).

To investigate the reason for the early mortality in *Baf60c^Myh6KO^* mice, their hearts were dissected at different ages for morphology and histology analysis. At all the observed stages (P10, P21 and 8 weeks), the hearts of *Baf60c^Myh6KO^* mice were larger than controls (Fig. 4B). Histology revealed chamber dilation (Fig. 4B). Masson's trichrome staining detected broad myocardium interstitial fibrosis in the *Baf60c^Myh6KO^* myocardium, while this was not observed in any other genotypes (Fig. 4B, lower panels). A high level of apoptosis was also detected in myocardium of adult *Baf60c^Myh6KO^* mice (Fig. 4C).

The chamber dilation and fibrosis observed in the hearts of *Baf60c^Myh6KO^* mice raised the question of whether cardiac function was also affected. We measured cardiac contractile function of 8-week-old mice that lacked outward signs of heart failure or growth delay, using high frequency echocardiography (Table 2, *n*=6). Confirming the histological results, the left ventricles of *Baf60c^Myh6KO^* mice were prominently dilated, and the anterior and posterior ventricle walls of *Baf60c^Myh6KO^* mice were thinner and the chamber contraction ratio decreased. The aortic time-velocity integral (TVI, which measures the distance traveled by a volume of blood during a time interval) increased, probably because of the enlarged ventricle volume. The fractional shortening (FS) and cardiac output were reduced, consistent with the cardiac failure symptoms of *Baf60c^Myh6KO^* mice. We performed electrocardiogram analysis to measure the conduction function of *Baf60c^Myh6KO^* mice (Fig. 4D, Table 3; *n*=5–6). Compared with other genotypes, *Baf60c^Myh6KO^* mice had significantly slower heart rates, shortened conduction time through the atrioventricular (AV) node (PR interval), and prolonged QRS duration, suggesting longer depolarization-repolarization time of the ventricle. P wave height, which indicates atrial depolarization, was reduced. Thus, clear and significant conduction defects accompany contractile deficiency in *Baf60c^Myh6KO^* mice.

**Table 2.**
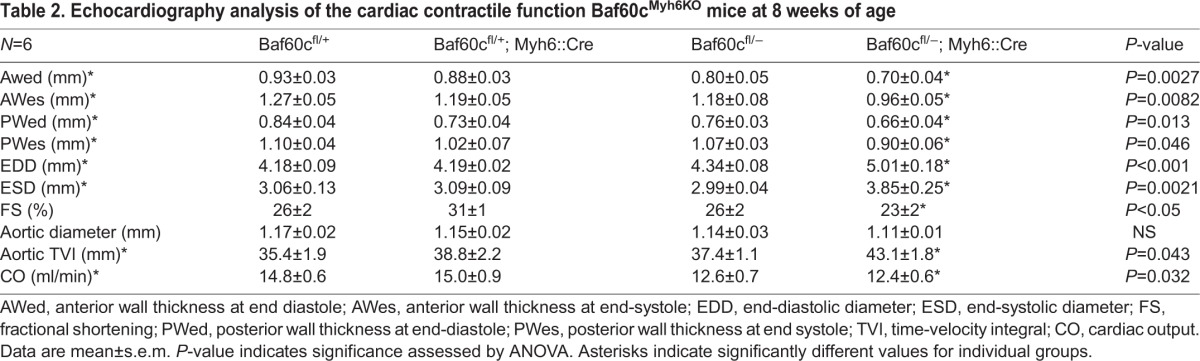
**Echocardiography analysis of the cardiac contractile function Baf60c^Myh6KO^ mice at 8 weeks of age**

**Table 3.**
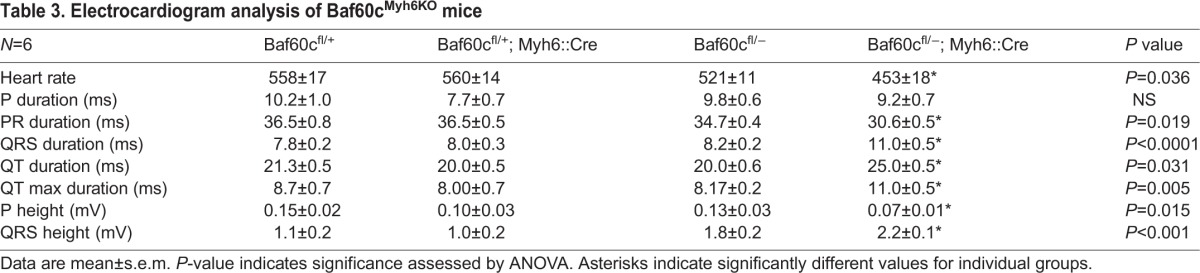
**Electrocardiogram analysis of Baf60c^Myh6KO^ mice**

### Myofibrillar defects of Baf60c KO cardiomyocytes

**Fig. 5.**
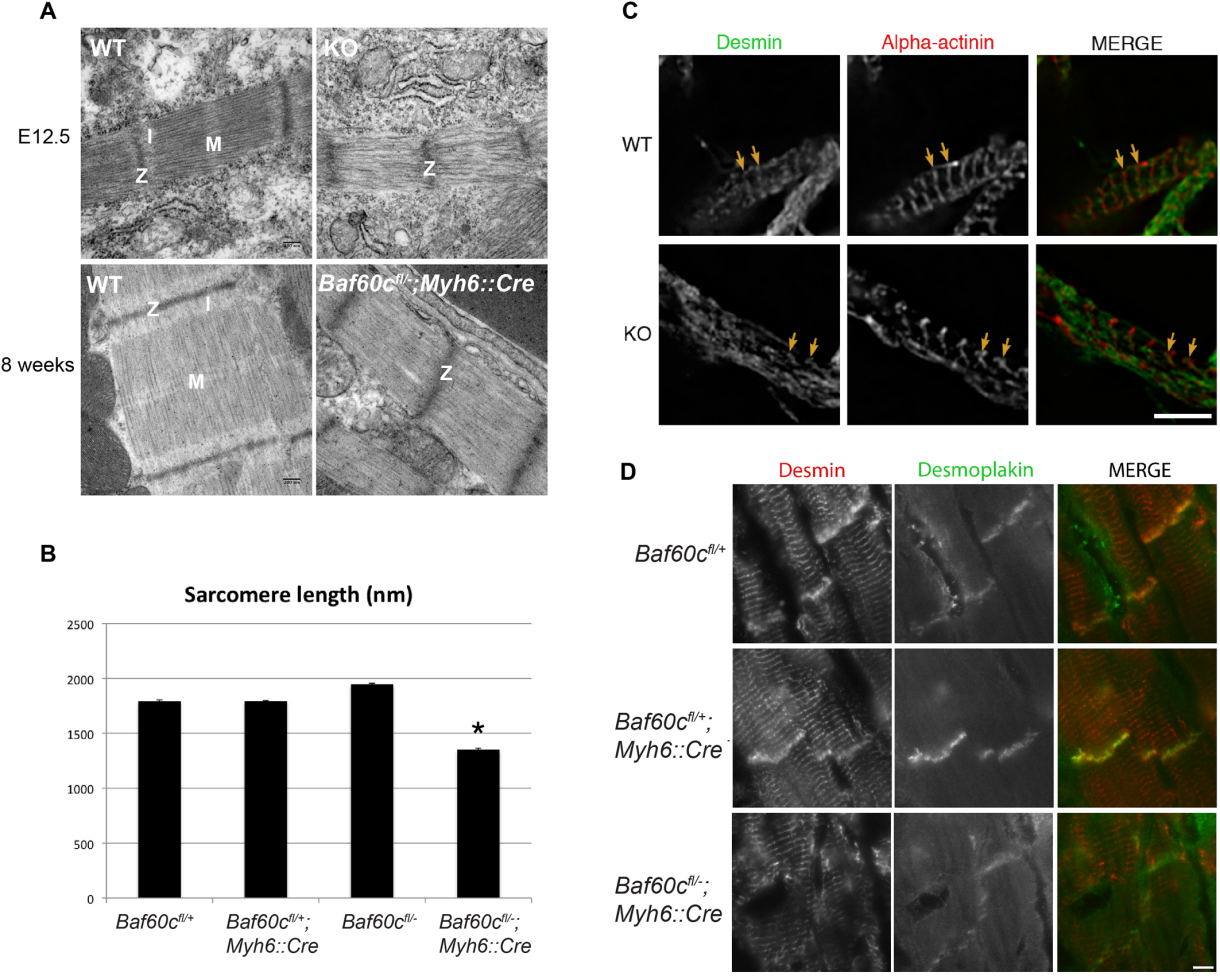
**Myofibrillar defects of *Baf60c*^−/−^ cardiomyocytes.** (A) Cardiomyocyte ultrastructure of WT and *Baf60c^−/−^* under Transmission electron microscopy (TEM). Z, Z-disk; I, I-band; M, M-line. (B) In adult mice, sarcomeres of *Baf60c^fl/−^;Myh6::Cre* sarcomeres are shorter. Mouse hearts were not relaxed before sample preparation, but only relaxing sarcomeres were measured. Error bars indicate s.e.m.; *P<0.05 by ANOVA test. (C,D) localization of Desmin was disturbed in embryonic and adult hearts in the absence of Baf60c. Yellow arrows indicate sarcomeres.

The cardiac structural and functional defects in *Baf60c^−^*^/*−*^ are a reflection of an underlying cellular defect. To address this, we used electron microscopy to observe cardiomyocyte ultrastructure. At E12.5, sarcomeres of *Baf60c^−^*^/*−*^ hearts were disarrayed, and the thick and thin filaments were discontinuous and poorly aligned. Z-disks were loosely packed and did not have clearly defined borders as in WT sarcomeres. The I bands (thick-filament free zone) and the M bands (myosin head free zone of the thick filaments) located in the middle of sarcomere were almost undetectable (Fig. 5A, top panel). Similar defects also existed in adult *Baf60c^Myh6KO^* cardiomyocytes. The sarcomere length in adult hearts (the length between two adjacent Z-disks) was significantly shorter in *Baf60c^Myh6KO^* mice than WT mice (Fig. 5B).

We examined the distribution of several important structural proteins in cardiomyocytes by immunofluorescence deconvolution microscopy, and found that the localization of Desmin in Z-disks of embryonic cardiomyocytes was disturbed in *Baf60c^−/−^* hearts (Fig. 5C). In adult *Baf60c^Myh6KO^* hearts, localization of Desmin in intercalated discs was also reduced (Fig. 5D), and the pattern of Desmin localization was perturbed (poorly aligned). These observations are similar to what was observed by electron microscopy and, together, show disrupted myofibril alignment and sarcomere structure in the absence of *Baf60c*.

### Cardiac gene expression program regulated by Baf60c

**Fig. 6.**
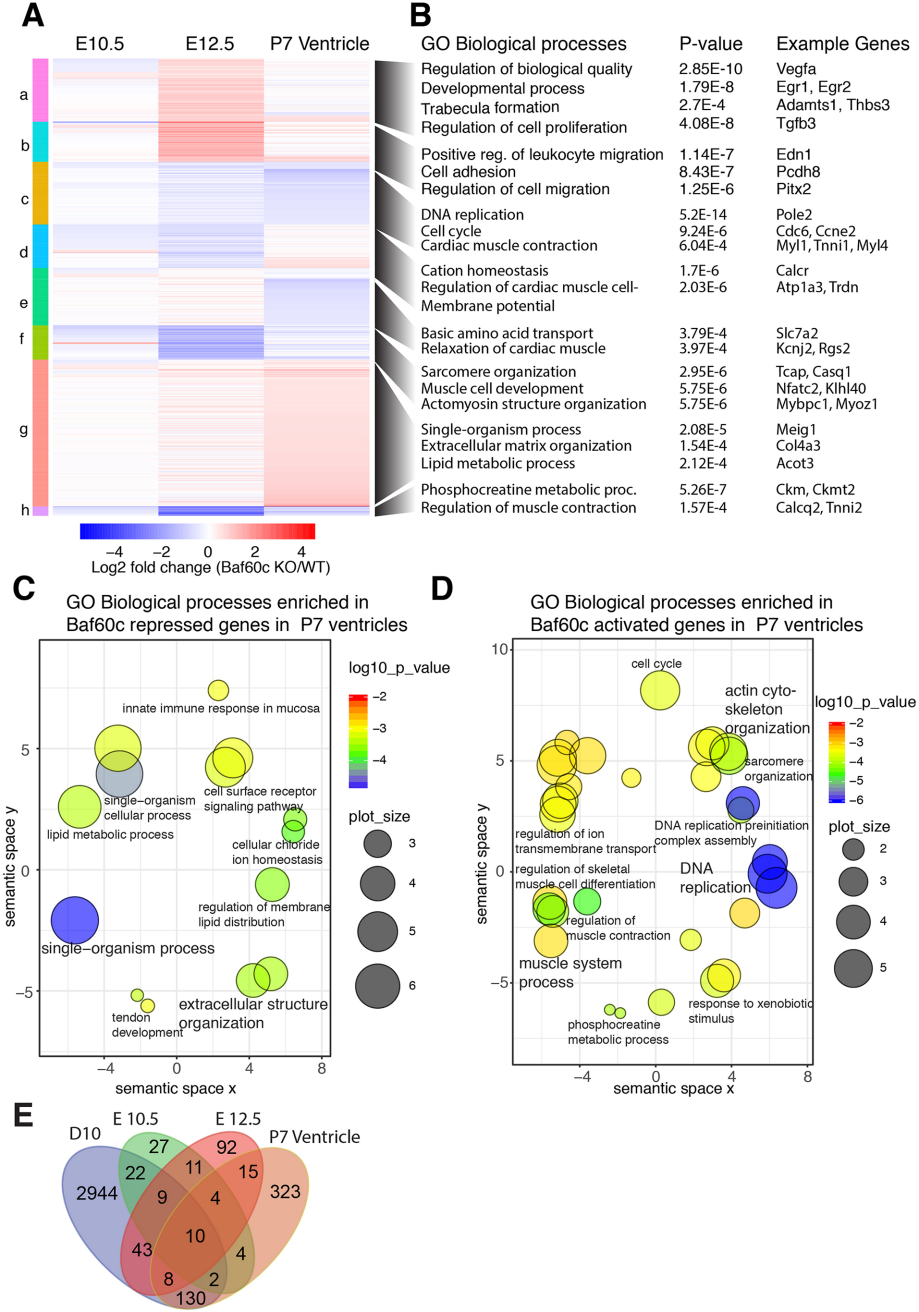
**BAF60c transcriptionally affects cardiac morphogenesis and function.** (A) Heat map comparing genes affected by *Baf60c* loss in embryonic hearts (E10.5 and E12.5) and postnatal ventricles [postnatal day (P)7]. Significantly affected (≥1.25-fold, *P*<0.05, Useq test, *n*=3) genes in at least any one stage were selected and clustered. (B) Gene ontology (GO) biological processes enriched in each of these clusters and example genes in that category are shown. (C,D) Genes repressed (C) or activated (D) by Baf60c in P7 ventricles were analyzed for enrichment of GO biological processes and are plotted. The color of circles represents *P*-value of enrichment and size represent the size of the GO term. (E) Venn diagram showing genes misregulated in absence of Baf60c in embryonic heart at E10.5, E12.5 and P7 ventricle and compared cardiac myocyte (Day 10) stages of *in vitro* directed cardiac differentiation.

To identify genes regulated by Baf60c, we used RNAseq to analyze total RNA prepared from *Baf60c^−^*^/*−*^ hearts and control hearts harvested at E10.5 and E12.5, and from P7 *Baf60c^Myh6KO^* and control hearts. We identified 788 genes that were differentially expressed by at least 1.25-fold (*P*<0.05) in at least one stage versus wild type (Fig. 6A; Supplemental Datasets 1 and 2). Among all the genes and all the analyzed stages, 132 genes were upregulated, and 175 were down-regulated at all time points. Misregulation of major cardiac transcription factors or signaling molecules was not observed. Instead, consistent with the ultrastructural findings, many genes related with cardiac metabolism and striated muscle contraction, such as *Acta1*, *Aldh1l2*, *Casq1*, *Casq2*, *Ckm*, *Ckmt2*, *Trim72*, *Kbtbd10 (Krp1)*, *Myh7b*, *Myl3*, *Mylpf*, *Obscn*, and *Tnni2*, were identified as downregulated in embryonic and adult *Baf60c*-deficient hearts (Fig. 6B). A broader range of cardiac function-related genes were deregulated in the *Baf60c^Myh6KO^* hearts, including *Gja3*, *Myl1*, *Myl4*, *Myl7*, and *Tnni1*. The postnatal deletion of *Baf60c* also resulted in induction of *Nppa*, as might be expected in a cardiomyopathic heart (Houweling et al., 2005), but the induction was mild (only twofold increase), indicating a potential deficiency in upregulation of this marker of cardiac stress. In fact, the usual cardiac stress-responsive genes were not present in the *Baf60c^Myh6KO^* cardiac gene expression program. Gene ontology (GO) analysis of genes repressed by *Baf60c* in postnatal heart enriched for biological processes involved in broad developmental processes and extracellular structure organization (Fig. 6C). However, *Baf60c*-activated genes were enriched for muscle system processes, regulation of muscle cell differentiation, muscle contraction, and sarcomere and actin cytoskeleton organization (Fig. 6D). An enrichment of cell-cycle-related genes was also apparent; it is not clear what this signifies and may reflect a role for *Baf60c* in regulating perinatal proliferation, which was not addressed in this study. These results collectively suggest that *Baf60c* is required for proper expression of genes encoding components or regulators of the contractile apparatus.

The analysis of gene expression in whole hearts has the disadvantage that a heterogeneous mix of cells may prevent clear identification of the full set of Baf60c-regulated genes, and also that some changes in gene expression may be secondary to altered hemodynamics. We compared the set of genes altered in the Baf60c mutant hearts with RNAseq analysis of cardiac precursors and cardiomyocytes differentiated *in vitro* from WT and *Baf60c*^−/−^ ES cells (Hota et al., 2017 preprint). Considerable overlap was found for the E12.5 KO hearts and ES cell-derived cardiac precursors differentially expressed genes, and more significant overlap was found for E12.5 KO and *Baf60c^Myh6KO^* hearts with ES cell-derived cardiomyocyte (Fig. 6E). These comparisons show that both *in vitro* and *in vivo*, *Baf60c* regulates a set of genes important for cardiac morphogenesis and function.

### BAF60c functionally interacts with Myocardin

**Fig. 7.**
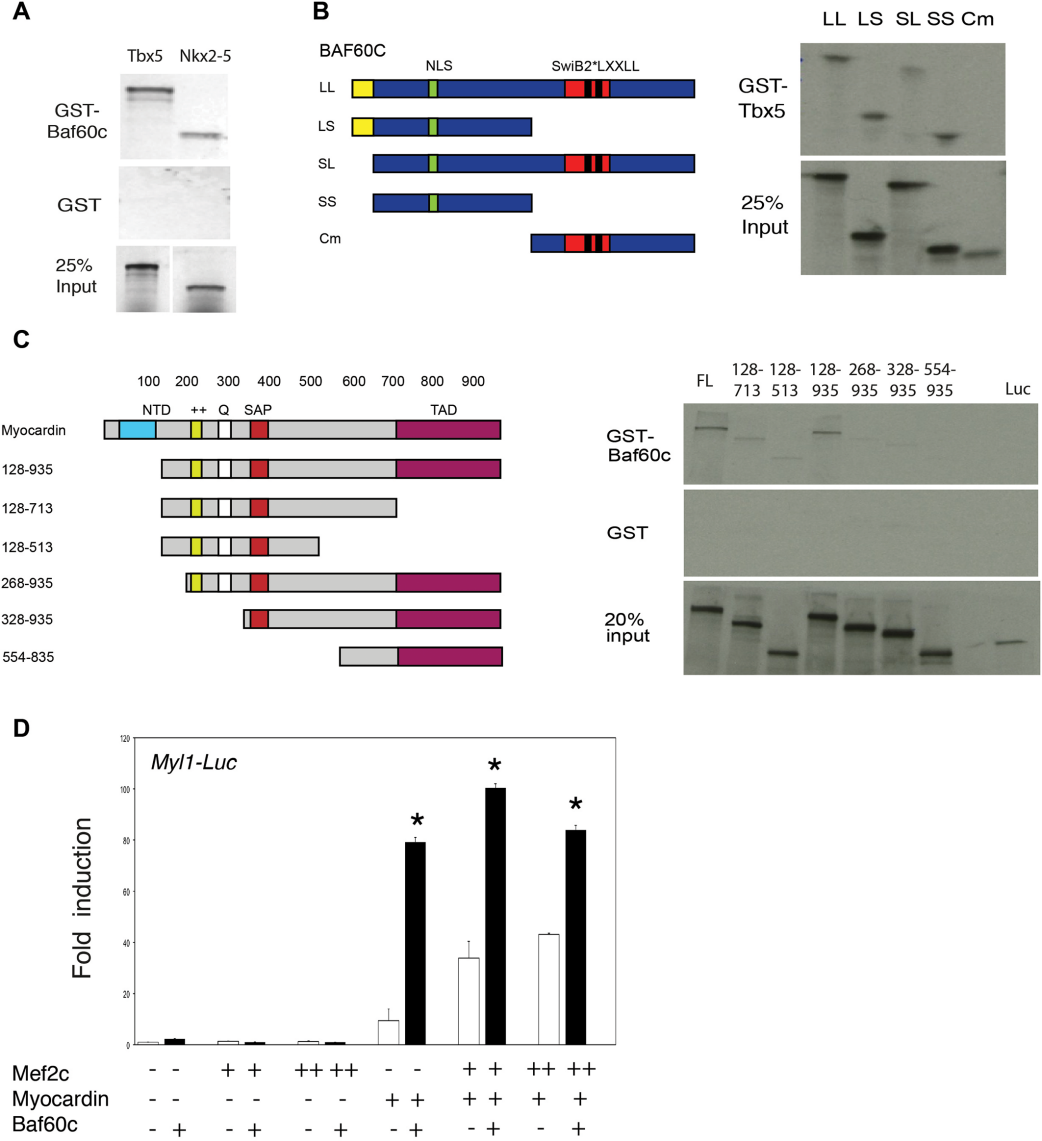
**Interaction between BAF60c and cardiac transcription factors.** (A) GST-fused Baf60c associates with ^35^S-labeled, *in-vitro* synthesized TBX5 and NKX2-5. (B) BAF60c associates with TBX5 through its N-terminal domain. Left: schemes representing serial deletion constructs of BAF60c. Right: mapping the BAF60c-associating domain with GST-fused TBX5. (C) BAF60c associates with full-length Myocardin and serial deletions. Left: schematic representation of Myocardin deletion constructs. The region at amino acids 328–554 of Myocardin was essential for association with Baf60c. (D) BAF60c enhances activation of MYOCD and MEF2c on the *Myl1* luciferase reporter. Data are mean±s.e.m. Asterisks indicate significantly different values (*P*<0.05 by ANOVA) for individual conditions by *t*-test.

We previously identified TBX5 and NKX2-5 as potential BAF60c-interacting proteins (Lickert et al., 2004). Here we used GST pulldown to show that these interactions can be direct (Fig. 7A). We mapped the BAF60c interaction domain to an N-terminal region that contains a nuclear localization signal sequence (Fig. 7B). To further elucidate the molecular mechanism of BAF60c function, we searched for potential association partners of BAF60c. In a yeast two-hybrid screen of a human heart cDNA library, using BAF60c as the bait, we identified few potential interacting factors (BAF155, FEZ1, MYOCD). BAF155 is a component of the BAF complex, which indicates a direct interaction between these two BAF complex subunits. Of particular interest among candidate interactors was Myocardin (MYOCD), a transcriptional co-factor of SRF and MEF2c (Creemers et al., 2006; Wang et al., 2001). A GST pull-down assay between GST-fused BAF60c and *in vitro* synthesized MYOCD confirmed the direct association, and mapped the association domain of MYOCD with BAF60c to amino acids 328–554 (Fig. 7C). *Myl1* is a bona fide direct target of MEF2c/Myocardin (Creemers et al., 2006) and was downregulated in the absence of *Baf60c*. In an *in vitro* promoter activation assay, BAF60c could potently enhance the activation of the *Myl1* promoter by MYOCD and MEF2c (Fig. 7D). We have not tested a genetic interaction between *Baf60c* and *Myocd*. Our data suggest that BAF60c functions as a partner of MYOCD in cardiac development, and that this interaction may be important for the activation of a gene expression program essential for the fundamental functional properties of cardiomyocytes.

## DISCUSSION

We showed the requirement for *Baf60c* in cardiomyocyte function throughout heart development. Loss of *Baf60c* both prenatally and postnatally resulted in cardiac hypoplasia and defective heart function. *Baf60c* regulates programs of gene expression that are essential for primary functions of cardiomyocytes, including broad sets of genes essential for sarcomere function and cardiac metabolism.

The *Baf60c* constitutive knockout phenotype is milder than the mouse shRNA knockdown phenotypes reported earlier (Lickert et al., 2004). The shRNA knockdowns used two independent shRNAs, minimizing the possibility of off-target effects, and the phenotype was rescued by over-expression of BAF60b, indicating significant specificity of the shRNAs. A similar discrepancy exists for *Ifitm* genes, for which the shRNA phenotype is more severe than that of a genetic deletion (Lange et al., 2008; Tanaka et al., 2005). The possible reasons for the different phenotypes between the shRNA and the genetic null might include effects compounding the loss of *Baf60c* function, such as overloading of the microRNA processing machinery by overexpressing shRNAs at high levels, other non-specific effects inherent to overexpression of shRNAs in the mouse embryo, or failure to compensate for immediate repression of gene function by RNAi. The genetic deletion here confirms an important role for *Baf60c* in heart development and extends these findings significantly.

The phenotype resulting from loss of *Baf60c* suggests that *Baf60c* has a specific role in regulating gene expression programs necessary for cardiac growth and contractile function. BRG1, the core ATPase of BAF complexes, has broad and critical roles in supporting cardiomyocyte proliferation and differentiation at embryonic stages and hypertrophic growth in the stressed adult heart (Hang et al., 2010; Takeuchi et al., 2011). Our data suggest that a BAF60c-containing cardiac-specific BAF complex has a more specialized role, and may have evolved to provide fine-tuned and specific gene regulation in the mammalian heart. Indeed, we isolated BAF complexes during *in vitro* cardiac differentiation, and found that BAF60c-containing complexes in cardiomyocytes have a composition that differs from many BRG1-containing complexes (Hota et al., 2017). In skeletal muscle differentiation, BAF60c interacts with MYOD to activate muscle-specific genes (Forcales et al., 2012), and is essential for HDAC-dependent fibro-adipogenic precursor differentiation in dystrophic muscle (Saccone et al., 2014). The set of genes that are altered due to depletion of BAF60c in differentiating C2C12 cells (Forcales et al., 2012) is remarkably similar to those altered by loss of BAF60c in the heart, indicating a commonality in the regulatory program controlled by BAF60c in cardiac and skeletal muscle. The role of BAF60c in glycolytic metabolism of fast-twitching muscle has also been described (Meng et al., 2013); whether Baf60c has a specific function regulating metabolic switching during cardiomyocyte maturation is a potential direction for future studies.

*Myocd* is an essential factor for embryonic cardiac gene expression and postnatal myocardial function (Creemers et al., 2006; Huang et al., 2012, 2009). Loss of *Myocd* in cardiac precursors results in a phenotype very similar to that of *Baf60c*-null embryos, with death around E13.5, thinned myocardium, ventricular septal defects, and reduced proliferation (Huang et al., 2012). Cardiomyocyte-specific deletion of *Myocd*, as with that of *Baf60c*, also results in sarcomere disorganization, mislocalization of Desmin, and apoptosis (Huang et al., 2009). However, the changes in gene expression documented in *Myocd*-deficient hearts are not fully recapitulated by the loss of *Baf60c*, indicating that a MYOCD/BAF60c interaction may target a specific subset of *Myocd*-regulated genes, such as *Myl1* and others. The association domain of MYOCD with BAF60c did not differentiate between the smooth muscle and cardiac isoforms (Creemers et al., 2006), suggesting BAF60c can either associate with both and regulate different programs, or there are other mechanisms *in vivo* controlling selective association with either isoform. BAF60c can act on SRF-dependent promoters to regulate smooth muscle gene expression (Sohni et al., 2012); it remains to be determined whether this activity involves an interaction with Myocardin.

Mutations in many cardiac transcription factor and structural genes result in congenital heart defects and cardiomyopathy (Ahmad et al., 2005; Bruneau, 2008; Fahed et al., 2013). Mutations in histone-modifying complex subunit genes and in some chromatin-remodeling protein-encoding genes have been identified in patients with congenital heart defects (CHDs) (Homsy et al., 2015; Zaidi et al., 2013). While no mutations in *SMARCD3*, which encodes BAF60c, have been associated with CHDs, the functional interaction of BAF60c with several transcription factors implicated in CHDs suggests that a potential underlying mechanism for CHDs may depend on BAF60c. Indeed, our recent proteomic analysis of BAF complexes identified WDR5, mutated in human CHD, as part of a cardiac-enriched BAF complex (Hota et al., 2017). In conclusion, we demonstrated the essential role of BAF60c in cardiac growth and function, and implied a possibility of chromatin-remodeling factors contributing to CHDs.

## MATERIALS AND METHODS

### ES cell targeting and mouse line establishment

A *Baf60c* genomic DNA fragment with loxP sites flanking 1st exon to 4th exon and Frt-Neo-Frt cassettes downstream of 4th exon was constructed using bacterial recombineering (Fig. 1A). For gene targeting, 5×10^6^ R1 ES cells were trypsinized and electroporated with 25 µg linearized targeting DNA. The electroporated cells were selected with 160 µg/ml G418 (Gibco # 10131) for 7 days. Correctly targeted clones were identified using Southern blots with DNA probes located outside the targeting DNA and labeled with ^32^P (Perkin Elmer). The clones were then expanded and used for diploid aggregation. High embryonic stem cell (ESC)-contributed chimera males were bred with ICR and C57/BL6 for germline transmission. *Baf60C^neo^*^/+^ progeny were mated with FLPe-expressing mice [B6;SJL-Tg(ACTFLPe)9205Dym/J, maintained at the Toronto Center for Phenogenomics (TCP), Canada] to remove the *Neo* cassette between the *frt* sites and yield *Baf60c^flox^*^/+^ mice. To generate the *Baf60c* deletion, *Baf60c^flox^*^/+^ mice were mated with pCX-NLS-Cre mice (maintained at the TCP). All animal work was carried out following Canadian Council on Animal Care Guidelines for Use of Animals in Research and Laboratory Animal Care under protocols approved by The Centre for Phenogenomics Animal Care Committee, and in accordance with the guidelines of the University of California, San Francisco (UCSF) Institutional Animal Care and Use Committee (IACUC).

### Mouse and embryo genotyping

The *Baf60c^flox^*^/+^ and *Baf60c*^+/−^ mice were genotyped by PCR using three primers: WT for (5′-CGTTCTGCAAGATGGTCTGA-3′), DEL for (5′-AGGCAGACCCAAGCTTGATA-3′) and Rev for (5′-CATCAGAGTCTTCCGCATCA-3′). Baf60c deletion band is 250 bp, wild type is 350 bp and Baf60cfloxed is 470 bp. Postnatal mouse tissues (tail tips or ear notches) and embryo tissues (yolk sac, tails, limb buds) were prepared with the tissue preparation buffer of the Sigma Extract-N-Amp tissue PCR kit (Sigma, XNAT2).

### Histology

Mouse embryos or tissues were fixed with 4% PFA, dehydrated and embedded with paraffin and sectioned into 4-µm sections then mounted on glass slides. The slides were then stained using standard histology protocols.

### Whole-mount *in situ* hybridization

Whole-mount *in situ* hybridization on mouse embryos at E7.5–10.5 was performed according to standard protocols with the *Baf60 in situ* hybridization probe (Lickert et al., 2004).

### Optical projection tomography

Optical projection tomography (OPT) was performed as described (Sharpe et al., 2002) with an OPT system built in-house. E12.5 embryos were harvested, genotyped, fixed with 4% PFA overnight and washed with PBS. The specimens were then embedded in 1% low-melting-point agarose and subsequently cleared using a 1:2 mixture of benzyl alcohol and benzyl benzoate. The index-matched specimen was suspended from a stepper motor and immersed in a benzyl benzoate bath encompassed in a glass cuvette. Light from a mercury lamp was directed onto the specimen and filter sets were used to create fluorescent images of the specimen. An autofluorescence projection was captured with using a GFP filter set in the illumination and detection light path. Images of the specimen were formed using a Qioptiq Telecentric Zoom 100 microscope equipped with a 0.5× OPTEM objective lens. Projection images were acquired with a Retiga-4000DC CCD camera with pixel size equal to 7.4 µm/pixel. The sample was rotated in finite steps, 0.3°, through a complete revolution totaling 1200 projections. Image reconstruction into a 3D data set was then executed by a modified Feldkamp algorithm in supplied software by SkyScan (NRecon, Bruker, Kontich, Belbium). The resultant OPT images have an isotropic 8.8 micron pixel size.

### RNA-seq

Mouse embryos from *Baf60c*^+/−^ intercross timed pregnancy at E10.5 and E12.5, or ventricles from *Myh6::Cre;Baf60c^fl^*^/+^ X *Baf60c*^+/−^ intercrosses were harvested. Both males and females were used. Their hearts were individually dissected and snap-frozen with liquid nitrogen. RNA was prepared from each single heart with the PicoPure RNA Isolation kit (Arcturus, Thermo Fisher, Waltham, MA, USA). RNA quantity and quality was analyzed using Agilent RNA 6000 Nano Kit. RNA-seq was performed as described (Christodoulou et al., 2011, 2014). RNA reads were aligned with TopHat/Bowtie (http://ccb.jhu.edu/software/tophat/index.shtml) and Useq (http://useq.sourceforge.net/) was used for the analysis of differential expression. RNAs that showed significant differential expression between wild type and *Baf60c*^−/−^ (*P*-value <0.05) and also changed more than 1.25-fold in *Baf60c*^−/−^ over wild type at a specific stage of differentiation were selected for analysis, avoiding duplicate and redundant entries.

### Transmission electron microscopy (TEM)

Mouse E10.5, E12.5 embryonic hearts and 8-week-old adult hearts (males and females) were dissected. For embryonic hearts, the whole heart was used for fixation and section. For adult hearts, pieces of 3∼4 mm in size cut from the left ventricle were used as specimens. Pieces of specimen were fixed in a fixative containing 4% formaldehyde and 1% glutaraldehyde in phosphate buffer, pH 7.3, and then post fixed in 1% osmium tetroxide. The specimens were then dehydrated in a graded series of acetone from 50% to 100% and subsequently infiltrated and embedded in Epon-Araldite epoxy resin. The processing steps from post fixation to polymerization of resin blocks were carried out in a microwave oven, Pelco BioWave 34770 (Pelco International, Redding, CA, USA) using similar procedures, with slight modification, as recommended by the manufacturer. Ultrathin sections were cut with a diamond knife on the Reichert Ultracut E (Leica, Vienna, Austria). Sections were stained with uranyl acetate and lead citrate before being examined in the JEM-1011 (JEOL USA, Peabody, MA, USA). Digital electron micrographs were acquired directly with a 1024 X1024 pixels CCD camera system (AMT, Danvers, MA, USA) attached to the TEM.

### Echocardiography assessment of cardiac functions

E13.5 embryos were analyzed with a Vevo770 ultrasound machine (VisualSonics, Toronto, Canada). Pregnant *Baf60c^+/−^* female mice carrying the embryos at the required developmental stages were examined under isoflurane anesthesia. Uteruses were exposed from the incision and scanned with a 30 MHz transducer as described (Lickert et al., 2004). To minimize potential impairment of embryonic physiology, only two or three embryos were scanned for each female, taking about 1 h. The mother's heart rate was monitored throughout the scanning. For each embryo, the blood flow speed near the mitral and tricuspid valves and aorta was recorded at B-mode. The depth of ventricle walls and ventricle septation was measured at M-mode. After scanning, the embryos were harvested and genotyped. Four to five embryos of each genotype were measured. Adult mice were analyzed using a Vevo2100 ultrasound machine (VisualSonics). The 7–8-week-old animals were anesthetized and scanned with a 30 MHz transducer as described (Zhou et al., 2005). E and A peaks in the left ventricle were measured at B-mode. The chamber dimensions and ventricle wall depths as well as ventricle septation depth were measured at M-mode. For each genotype, five or six mice were measured.

### Electrocardiography

Mice (male, 8 weeks old) were anesthetized with 1–2% isoflurane, and a lead II ECG was recorded from needle electrodes inserted subcutaneously into the right forelimb and into each hind limb. The signal was recorded for ∼1 min. The ECG was recorded with Power Lab/4SP (AD Instruments, Dunedin, New Zealand) and analyzed using the SAECG (signal-averaged electrocardiogram) extension for Chart 4 (v4.2.3 for Macintosh, AD Instruments).

### Immunofluorescence microscopy

Sarcomeric architecture and organization were assessed in E12.5 and adult hearts via double immunofluorescence staining. Heart tissue was embedded in Tissue-Tek Optimum Cutting Temperature (OCT) compound (Sakura Finetek) and immediately frozen in 2-methylbutane precooled in liquid nitrogen. 5-mm cryosections were mounted on gelatin-coated glass coverslips. Tissue sections were fixed in 4% paraformaldehyde, permeabilized with 0.2% Triton-X 100/PBS and blocked with 2% BSA/1% normal donkey serum/PBS prior to incubation with antibodies. The primary antibodies included: rabbit polyclonal anti-desmin (1:30) (ImmunoBioscience RP-4023-04), mouse monoclonal anti-sarcomeric a-actinin (1:1000) (Clone EA-53; Sigma A7811), and mouse monoclonal anti-desmoplakin 1/2 (1:1000) (Clone DP-2.15; AbDSerotec 2722-5204) antibodies. The secondary antibodies, obtained from Jackson Immunoresearch Laboratories, included Alexa Fluor 488 goat anti-mouse IgG (1:500), Alexa Fluor 488 goat anti-rabbit IgG (1:500), Texas Red goat anti-mouse IgG (1:500), and Texas Red goat anti-rabbit IgG (1:500). Coverslips were mounted onto slides with Aqua Poly/Mount (Polysciences Inc., Warrington, PA, USA). All sections were analyzed on a Deltavision RT system with 100× (1.3 NA) objective and a CoolSnap HQ charge-coupled device camera (Photometrics, Tucson, AZ, USA) using softWoRx 3.5.1 software. Images were prepared for presentation using Photoshop CS (Adobe Systems).

### TUNEL analysis

Cell death on sections was detected using Roche In Situ cell death detection kit Fluorescein (11684795910).

### Yeast two-hybrid assay

A full-length BAF60c expression construct was used as a bait in a yeast two-hybrid assay conducted by Hybrigenics (www.hybrigenics-services.com/), using a human fetal/adult heart library.

### Luciferase assay

The *Myl1* luciferase construct was as described (Creemers et al., 2006). Combined DNA vectors were transfected into early exponential stage 10T1/2 cells cultured in six-well dishes with Fugene 6 (Roche, 1181443001), following the product manual. After culturing for another 40–48 h, the cells were lysed, and luciferase activity analyzed with Dual-Luciferase Reporter Assay System (Promega E1910). The luciferase activity was normalized with renilla activity. Three biological replicates were prepared for each combination.

### GST-pulldown assay

^35^S-labeled proteins (TBX5, NKX2-5, RBPjk, NICD, BAF60c serial deletions, Myocardin serial deletions) (Wang et al., 2001) were synthesized with the TnT SP6 coupled reticulocyte lysate system (Promega, L4600) or TnT T7 coupled reticulocyte lysate system (Promega L4610) and labeled with ^35^S methionine (Perkin Elmer NEG709A). 5 µl of each synthesized protein was analyzed with SDS-PAGE gel and exposed to X-ray film for evaluation. GST-BAF60c, GST-RBPjk, GST-TBX5 and GST were expressed in *E. coli* strain BL21 and purified with glutathione Sepharose 4B (GE Healthcare, 17-0756-01). The beads were incubated with ^35^S-labeled target proteins overnight at 4°C and washed with PBST for three times. The beads were then boiled in loading buffer. The protein was analyzed with SDS-PAGE gel and autoradiography.

### Statistics

Data were expressed as mean±s.e.m. Differences among multiple experimental groups were evaluated by ANOVA followed by post hoc Fisher's LSD test. Pairwise comparisons were evaluated by unpaired two-tailed Student's *t*-tests. *P*<0.05 was considered as significant.
